# Leachates of Calcium-Rich Phases from Attapulgite Clay as a Sustainable Calcium Source for Microbially Induced Carbonate Precipitation: Enhanced Biomineralization and Arsenic Immobilization

**DOI:** 10.3390/toxics14080678

**Published:** 2026-07-31

**Authors:** Lei Wang, Xiang Ning, Meng Yang, Shengli Wang

**Affiliations:** 1 Gansu Nonferrous Engineering Survey Design and Research Institute, Lanzhou 730000, China; 2 College of Earth and Environmental Sciences, Lanzhou University, Lanzhou 730000, China

**Keywords:** microbial induced carbonate precipitation (MICP), attapulgite, calcium source, biomineralization, arsenic immobilization, soil remediation

## Abstract

Microbially induced calcium carbonate precipitation (MICP) is a promising biotechnology for environmental remediation; however, the high cost of conventional chemical-grade calcium sources limits its large-scale scalability. This study evaluated the feasibility of utilizing an aqueous extract of natural attapulgite clay as a sustainable, low-cost calcium source for MICP-mediated arsenic (As) immobilization in both aqueous and soil systems. Among the tested minerals, Baiyin attapulgite (group B) exhibited the highest calcium content (62,808.94 mg kg^−1^) and minimal toxic metal impurities, providing a favorable chemical matrix for biomineralization. At an optimal solid-to-liquid ratio of 1:10, *Lysinibacillus fusiformis* LF and *Enterococcus* LZU-1 successfully induced calcite precipitation driven by the attapulgite extract. In batch aqueous remediation experiments (20 days), the attapulgite extract significantly enhanced As removal efficiency compared to the controls; As removal rates peaked at 66.4% for strain LZU-1 (with LZ1 extract) and 65.8% for strain LF (with group B extract), drastically outperforming the standard CaCl_2_ groups (31.2–37.3%) and blank controls (21.8–24.5%). Concurrently, soil incubation experiments (30 days) demonstrated that the combined application of attapulgite and MICP bacteria reduced the highly bioavailable exchangeable As fraction from 0.115 to approximately 0.03 mg kg^−1^, while effectively driving its transformation into more stable carbonate-bound and organic-bound fractions without causing secondary soil salinization. Morphological and mechanistic analyses revealed that, compared to the well-defined euhedral crystals in the CaCl_2_ control, the precipitates mediated by the clay extract exhibited distinctly irregular, defect-rich rhombohedral structures. This structural disruption was governed by the natural matrix effect of attapulgite, which simultaneously supplied dissolved Ca^2+^ and provided an abundance of fine clay fragments, calcite micro-grains, and associated amorphous Fe/Al/Mn-bearing phases. These constituents acted as physical scaffolding and heterogeneous nucleation sites that became embedded in the growing CaCO_3_ lattice, driving the formation of highly reactive, defect-rich clay-calcite-arsenic composite precipitates that efficiently encapsulated arsenate. Mantel analysis further revealed that the remediation efficiency was significantly correlated with key environmental variables including Ni, V, Ca. These findings highlight the dual-system potential of natural attapulgite as an inexpensive, eco-friendly calcium alternative for sustainable MICP-based remediation of As-contaminated water and agricultural soils.

## 1. Introduction

Arsenic (As) is a naturally occurring metalloid that is widely distributed throughout the Earth’s crust, as well as in air, water, and soil. As a highly toxic metalloid, As poses a significant threat to global public health and food safety through its accumulation in the food chain [1]. In natural environments, soil and groundwater serve as two major and interconnected reservoirs of As [2]. The intensification of industrial and agricultural activities has led to widespread As contamination of soils worldwide [3]. In China, the average As concentration in soils increased from 11.9 mg kg^−1^ in 2000 to 12.6 mg kg^−1^ in 2020 and is projected to reach 13.6 mg kg^−1^ by 2040 [4]. Compared with the environmental background value of As in Chinese soil at 9.6 mg kg^−1^, the rate of soil exceeding the standard has been increasing year by year. Therefore, environmentally friendly and sustainable remediation strategies are urgently needed to restore contaminated soils, improve soil health, and promote sustainable agricultural production. However, existing remediation technologies often suffer from high costs, limited long-term stability, or adverse environmental impacts, highlighting the need for sustainable alternatives.

Advanced biotechnological approaches, particularly bioremediation, have emerged as cost-effective and environmentally sustainable remediation strategies [5]. Interactions between minerals and microorganisms have long played a fundamental role in shaping the Earth’s lithosphere [6]. During microbiologically induced calcite precipitation (MICP), ureolytic bacteria catalyze the hydrolysis of urea, producing carbonate and ammonium ions that drive calcite formation [7]. The released ammonium increases the surrounding pH, creating favorable conditions for the reaction between calcium and carbonate ions and thereby promoting calcite precipitation [8,9]. The remediation performance of MICP is influenced by multiple factors, including microbial species, nutrient availability, urea sources, heavy metal characteristics, and environmental conditions. MICP stabilizes heavy metals by transforming bioavailable fractions into carbonate-bound and residual fractions, thereby substantially reducing their ecological toxicity [10]. Furthermore, crystal densification and lattice incorporation during biomineralization enhance the long-term immobilization of various heavy metals [11]. Despite its considerable potential, the large-scale application of MICP remains constrained by the high cost of conventional reagents, particularly chemical-grade calcium sources such as CaCl_2_ [12]. The dependence on purified calcium salts substantially increases operational costs and limits the scalability of MICP for practical environmental remediation [13]. Consequently, increasing attention has been devoted to identifying low-cost and sustainable alternative calcium sources for MICP [14]. Recent studies have investigated a variety of waste-derived and agricultural calcium sources, including oyster shells, eggshells, scallop shells, agricultural by-products, and industrial effluents [15,16,17,18,19]. Although these alternative calcium sources have demonstrated promising feasibility, many require labor-intensive pretreatment processes or pose potential risks of secondary contamination, thereby limiting their practical application.

Attapulgite (also known as palygorskite) is a naturally occurring fibrous magnesium–aluminum phyllosilicate clay mineral with the chemical formula (Mg,Al)_2_Si_4_O_10_(OH)·4H_2_O [20]. China possesses abundant attapulgite reserves, particularly in Gansu Province, where extensive mining and processing activities generate substantial quantities of clay residues and associated wastewater. Its abundant availability, low cost, high adsorption capacity, excellent thermal stability, high surface activity, and strong ion-exchange capacity make attapulgite an attractive material for environmental applications. Its unique nanoporous structure enables attapulgite to effectively improve the physicochemical properties of environmental substrates [21]. Macronutrients and micronutrients released from attapulgite can enhance microbial growth and functional activity [22,23]. The low-grade attapulgite in Gansu region is characterized by complex accompanying minerals (such as muscovite, quartz, calcite, etc.). Attapulgite naturally contains exchangeable cations, including calcium and magnesium, which can be readily released through simple water leaching [24]. Furthermore, its high specific surface area and strong adsorption capacity make attapulgite a promising material for environmental remediation [25,26]. Based on these characteristics, we hypothesized that attapulgite could serve as a sustainable calcium source for MICP, thereby reducing the reliance on expensive chemical-grade calcium reagents. In addition, trace elements released from attapulgite may enhance microbial metabolic activity and functional performance under As stress, thereby improving the efficiency of bioremediation.

However, the potential of attapulgite as a sustainable calcium source for MICP and its synergistic role in As immobilization have not yet been systematically investigated. We hypothesized that attapulgite could simultaneously supply bioavailable calcium for biomineralization and enhance As immobilization. Accordingly, the objectives of this study were to (1) investigate the feasibility of using attapulgite as an alternative calcium source for MICP and elucidate its role in the biomineralization process, and (2) evaluate the effectiveness of attapulgite-derived calcium in promoting As immobilization in contaminated soils. This work provides a scientific basis for the resource utilization of attapulgite and offers a sustainable, low-cost strategy for the remediation of As-contaminated soils.

## 2. Materials and Methods

### 2.1. Determination of Physicochemical Properties of Attapulgite

Four attapulgite clay samples were obtained from Gansu Province, China: Baiyin attapulgite (B), and three Linze attapulgite samples (LZ1, LZ2, LZ3). The collected clay samples were air-dried naturally, ground, and passed through a 0.15 mm sieve before use [27]. Attapulgite pH was determined using the electrode method with a water-to-soil ratio of 5:1. A 3 g soil sample (sieved through a 2 mm mesh) was weighed into a 50 mL centrifuge tube, and 15 mL of CO_2_-free distilled water (prepared by boiling distilled water for 10 min, then sealing and cooling to room temperature) was added. The mixture was shaken thoroughly and allowed to stand for 30 min. The bulb of the pH meter (FE28, Mettler; Beijing United Ke-Yi Technology Co., Ltd., Beijing, China) was fully immersed in the supernatant, and the reading was recorded once it had stabilized. Attapulgite electrical conductivity (EC) was also determined using the electrode method with a water-to-soil ratio of 5:1. A 3 g soil sample (sieved through a 2 mm mesh) was weighed into a 50 mL centrifuge tube, and 15 mL of distilled water was added. The sample was shaken for 30 min at 25 °C and 220 rpm, centrifuged at 3000 rpm for 10 min, and the EC value was measured using a conductivity meter (DDSJ-308F, Shanghai INESA Scientific Instrument Co., Limited company, Shanghai, China). Sieving through a 2 mm mesh isolates the chemically active “fine earth” fraction, ensures a highly homogenous and representative 3 g sample, and protects the fragile glass pH electrode from physical damage. Acid-base neutralization titration was used for determination the content of calcium carbonate (CaCO_3_): 3.0 g of air-dried, sieved (0.149 mm) attapulgite was weighed into a 100 mL beaker, and 20.00 mL of 0.5 mol/L HCl standard solution (standardized against borax or NaOH) was added. The mixture was stirred with a glass rod to expel generated CO_2_. After cooling, the mixture was transferred to a 100 mL volumetric flask; the beaker was rinsed five times with cold water, and the flask was filled to the mark with water and shaken well. After allowing the suspension to settle, 50 mL of the supernatant was pipetted into a 150 mL conical flask, and two drops of phenolphthalein indicator were added. The solution was then titrated with NaOH standard solution (standardized against potassium hydrogen phthalate) until a distinct red color appeared, marking the endpoint. In short, using a 0.149 mm sieve ensures that CaCO_3_ particles are fully exposed for complete acid dissolution, breaks down protective organic/clay coatings, and homogenizes the sample to prevent massive errors caused by uneven carbonate distribution.

### 2.2. Bacterial Strains and Culture Conditions

Two ureolytic bacterial strains were employed in this study: (1) *Lysinibacillus fusiformis* LF (BNCC 210664), obtained from the BeNa Culture Collection (BNCC) (Beijing, China); (2) *Enterococcus* LZU-1 (CGMCC 22622), isolated and preserved at the China General Microbiological Culture Collection Center (CGMCC) [28] (Figures S1 and S2). Bacterial strains were maintained on nutrient broth (NB) agar plates and subcultured monthly. For experimental use, single colonies were inoculated into 50 mL of NB medium (peptone 10 g/L, beef extract 3 g/L, NaCl 5 g/L, pH 7.0 ± 0.2) and incubated at 30 °C with shaking at 150 rpm until the optical density at 600 nm (OD_600_) reached approximately 1.0.

The liquid medium for MICP experiments was prepared by dissolving peptone (10 g), NaCl (5 g), and beef extract (3 g) in 1000 mL of attapulgite aqueous extract. The pH was adjusted to 6.8 ± 0.2, and the medium was sterilized by autoclaving at 121 °C for 20 min. For precipitation experiments, the bacterial culture (1 mL, OD_600_ ≈ 1.0) was inoculated into the attapulgite extract-based medium supplemented with urea at a final concentration of 6% (*w*/*v*). The cultures were incubated at 30 °C with shaking at 150 rpm for 3–5 days. Control experiments were performed using conventional CaCl_2_ (60 mM) as the calcium source under otherwise identical conditions. Blank controls without bacterial inoculation were also included [29].

The white precipitates formed during MICP were collected by centrifugation, washed with distilled water, and freeze-dried. The morphology of the precipitates was examined using scanning electron microscopy (SEM) (Apreo S, Thermo Fisher Scientific, Waltham, MA, USA). The mineralogical composition was determined by X-ray diffraction (XRD) with Cu Kα radiation (λ = 1.5406 Å) over a 2θ range of 5–80° (SmartLab SE, Rigaku Corporation, Tokyo, Japan).

### 2.3. Attapulgite Extraction Procedures and Aqueous As Remediation via MICP

Attapulgite clay samples were mixed with distilled water at a solid-to-liquid ratio of 1:10 (m/V) and shaken in a thermostatic oscillator at 25 °C and 200 rpm for 2 h. The suspension was then centrifuged at 4000 rpm for 20 min, and the supernatant was filtered to obtain the attapulgite aqueous extract. For solid-to-liquid ratio optimization experiments, ratios of 1:2, 1:5, and 1:10 were tested. The extract was acidified with 0.5 M HCl to eliminate carbonate interference: acidification converts dissolved carbonates into CO_2_, preventing CaCO_3_ precipitation and ensuring accurate calcium determination by FAAS (SOLAAR M6, Thermo Scientific, Waltham, MA, USA). Based on this method, we determined the contents of calcium, magnesium, iron and manganese in the extract.

Three distinct types of attapulgites were utilized as natural material bases to induce MICP for As remediation from aqueous environments. To prepare the aqueous extract: 35 g of B and LZ1, LZ2 attapulgite clay powder was suspended in distilled water at a fixed solid-to-liquid ratio of 1:10 (*w*/*v*) to achieve a total volume of 350 mL. The mixture was continuously agitated in an orbital shaker at 200 rpm and 25 °C for 2 h to ensure efficient dissolution and extraction of soluble mineral components. Subsequently, the crude suspension was centrifuged at 4000 rpm for 10 min, and the supernatant was separated. The supernatant was then filtered to collect 300 mL of clarified attapulgite clay aqueous extract, which was stored under sterile conditions prior to use.

Batch As removal experiments were conducted using 50 mL conical flasks arranged in triplicate. Two distinct bacterial strains, designated as LZU-1 and LF, were evaluated for their respective MICP efficiencies. The experimental groups were categorized as follows: Blank Control Group (CK): Prepared using distilled water, comprising a nutrient broth matrix containing a final As concentration of 20 mg/L (supplied via Na_3_AsO_4_), supplemented with 6% (*w*/*v*) urea and inoculated with 1 mL of the enriched bacterial culture (either LZU-1 or LF strain) without any external calcium supplementation. Standard Control Group (CaCl_2_): Prepared using distilled water, featuring the identical nutrient broth-As matrix (20 mg/L As) and 6% urea, but supplemented with 60 mM of CaCl_2_ as a conventional chemical calcium source, followed by 1 mL bacterial inoculation. Experimental Attapulgite Groups (B, LZ1, and LZ2): Prepared by substituting distilled water with the respective attapulgite clay aqueous extracts as the sole calcium and material source. The medium consisted of the extract, nutrient broth, 20 mg/L As, and 6% urea, inoculated with 1 mL of the enriched bacterial culture.

All liquid nutrient media containing As and nutrient broth were sterilized by autoclaving at 121 °C for 20 min and allowed to cool to room temperature. Under aseptic conditions within a biosafety cabinet, 1 mL of the respective enriched bacterial strain (LZU-1 or LF) was inoculated into each flask. Concurrently, a stock solution of urea was sterilized using a 0.22 μm aqueous-phase membrane filter and added to the flasks to yield a definitive final concentration of 6% (*w*/*v*). The bioreactors were incubated in a temperature-controlled shaking incubator at 25 °C and 150 rpm for a continuous duration of 20 days. Upon completion of the incubation period, sub-samples were collected to determine the residual soluble As concentration.

### 2.4. Determination of All Elements of Attapulgite

Weigh 0.1 g (accurate to 0.0001 g) of attapulgite sample, air-dried and ground to a particle size less than 0.149 mm, into a digestion vessel. Add 1 mL hydrochloric acid, 4 mL nitric acid, 1 mL hydrofluoric acid, and 1 mL hydrogen peroxide. Place the digestion vessel in a microwave digestion system and heat the sample to 175 °C within 10 min, then maintain this temperature for 20 min. After digestion, cool the vessel to room temperature, carefully open the lid, and place it in an acid evaporation apparatus. Evaporate the acid at 150 °C under open conditions until the contents are nearly dry. Cool to room temperature, dissolve the residue with 2% nitric acid solution, transfer the solution to a 50 mL volumetric flask, and dilute to the mark with 2% nitric acid solution. Take the supernatant for analysis by an inductively coupled plasma mass spectrometer (ICP-MS) (ELAN DRC-e, PerkinElmer, Waltham, MA, USA). To prepare the blank sample, follow the same procedure without weighing any sample, ensuring that the amount of acid added to the blank matches that used in the sample preparation.

w=((ρ∗f−ρ_0)∗V)/(m∗w_dm∗1000)

The content of the target element in the attapulgite sample, denoted as w (mg/kg), is calculated based on the following parameters: ρ represents the mass concentration of the target element in the test sample (µg/L), ρ_0 is the mass concentration of the corresponding target element in the blank sample (µg/L), *V* signifies the constant volume of the sample after digestion (mL), f is the dilution factor of the sample, *m* denotes the mass of the attapulgite sample weighed (g), and w_dm indicates the dry matter content of the soil sample (%) with a scaling factor of 1000. The analyzed sample contains a suite of elements including selenium (Se), cobalt (Co), nickel (Ni), copper (Cu), molybdenum (Mo), cadmium (Cd), aluminum (Al), As, boron (B), barium (Ba), calcium (Ca), chromium (Cr), iron (Fe), potassium (K), lanthanum (La), lithium (Li), magnesium (Mg), manganese (Mn), sodium (Na), phosphorus (P), lead (Pb), sulfur (S), scandium (Sc), strontium (Sr), titanium (Ti), vanadium (V), and zinc (Zn).

### 2.5. Composition Analysis of Attapulgite Clay

In this study, X-ray diffraction (XRD) phase analysis was employed to quantitatively determine the mineral composition of attapulgite clay. The analysis was conducted by the Northwest Institute of Eco-Environment and Resources, Chinese Academy of Sciences. The attapulgite clay samples were air-dried, ground, and sieved through a 0.075 mm mesh before mineral composition analysis using an X-ray diffractometer (Cu Kα radiation; operating conditions: 40 kV, 30 mA) [30]. Sieving attapulgite clay through a 0.075 mm mesh is essential to randomize crystallite orientation, optimize diffraction intensity statistics, eliminate microabsorption, and provide a perfectly flat surface required for precise, quantitative XRD mineral identification. Qualitative and quantitative analyses were performed through spectrum interpretation. Whole-rock and clay mineral compositions were determined via scanning analysis. The specific procedure involved two main steps: clay extraction and clay separation. A 20 g powder sample was placed in a 250 mL beaker, and tap water was added to the 150 mL mark; the mixture was stirred thoroughly to suspend the clay minerals. Hydrochloric acid was added until gas evolution ceased, and the mixture was allowed to settle for 4 h until the supernatant appeared uniformly turbid. A syringe was used to extract the suspension from the upper third of the dispersed liquid, which was then transferred to a centrifuge tube. If non-uniform turbidity persisted in the upper part of the beaker after the initial washing, a special treatment (ultrasonic dispersion) was applied: a few drops of 5% sodium hexametaphosphate solution were added, followed by soaking for 5–10 min; ultrasonic dispersion was then applied for approximately 3 min, and the mixture was allowed to settle for 4 h prior to extraction. The extracted suspension was centrifuged (3500 r/min for 10 min) to sediment the clay particles; the supernatant was removed, and the clay residue was retained for the preparation of oriented mounts. Attapulgite were air-dried, sieved through a 2 mm mesh, and analyzed using a Malvern Mastersizer 2000 laser (Malvern Mastersizer 2000, Malvern Panalytical, Shanghai, China) particle size analyzer.

### 2.6. As Immobilization in Soil

In order to study the effect of attapulgite on the remediation of As by MICP, we collected As-contaminated soil and conducted indoor culture experiments. Heavy metal contaminated soil was collected from the farmland soil in Luojia Tan Village, Baiyin City, Gansu Province (36°32′24.00″ N, 104°18′24.02″ E). The total content of As was 200.86 mg/kg, which exceeded the screening value for agricultural land soil pollution (30 mg/kg), and it was classified as a mild pollution [31]. The specific operation steps were as follows: firstly, the soil was screened by a 2 mm sieve, and then the blank treatment was to weigh 14 g soil samples and put them into a 50 mL centrifuge tube, and add 6 mL distilled water (CK); Adding MICP bacteria only: Weigh 14 g soil sample, add 3 mL LF enriched bacteria solution (OD_600_ value is about 1.0) and 3 mL distilled water (U); Attapulgite addition: 14 g of contaminated soil is weighed and mixed with 10% B-attapulgite evenly, and 6 mL of distilled water is added (AT); MICP bacteria and attapulgite were added together: 14 g of contaminated soil was weighed and evenly mixed with 10% attapulgite, 3 mL of LF enriched bacteria solution and 3 mL of distilled water were added (ATU), and the experiment was finished after passivation for 30 days, during which distilled water was added to keep the water content the same and passivated in the same external environment [32].

Destructive sampling was used to obtain the restored soil. After natural air drying, the pH and EC values were determined according to the electrode method. A modified Tessier’s five-step continuous extraction method was used to extract different forms of As in the soil, including exchangeable state (F1), carbonate bound state (F2), iron-manganese oxidized bound state (F3) and organic bound state (F4), to analyze the mechanism of the remediation process. The highly stable residual fraction (F5) was excluded from this analysis as it is environmentally inert and does not participate in short-term passivation processes. MICP passivation of As in soil mainly transforms the exchangeable state (F1) of As in soil into carbonate bound state (F2) and other chemically more stable forms, thus reducing its bioavailability and mobility in soil and achieving the purpose of stabilizing it. Exchangeable state is the most active existing form, which is easy to migrate and transform in the environment and be absorbed and utilized by plants. Therefore, reducing the exchangeable content is of great significance to control the enrichment of heavy metals in the food chain and prevent groundwater pollution. The passivation rate of this study is the reduction rate of exchangeable state [26]. The calculation method is as follows:

$$\eta =\frac{C_{0}-C_{i}}{C_{0}}\times 100$$

where η is the passivation rate of As repair, %; C0 is the exchangeable metal concentration in the soil of the control group, mg/kg, after only adding NB culture solution and the same treatment as the experimental group; Ci is the exchangeable heavy metal concentration in the soil treated by attapulgite clay or MICP, mg/kg.

### 2.7. Statistical Analysis

All analysis was carried out in R software (version 4.3.2). In addition, a Tukey’s Honestly Significant Difference (HSD) was carried out to further explore the differences of these variables among different treatment groups (CK, U, AT, ATU). At the same time, the Spearman correlation coefficient was calculated to evaluate the relationship between the efficiency of attapulgite remediation and the As remediation by MICP and attapulgite and the trace and macro elements of attapulgite and the mineral composition of attapulgite clay.

## 3. Results

### 3.1. Characteristics of Macro-Elements and Trace Elements of Attapulgites

The concentrations of the macro-elements Al, Ca, Fe, K, Mg, Mn, Na, P, S, and Ti exhibited distinct group-specific distribution patterns. Al and Fe were the dominant matrix elements, with both reaching their highest concentrations in group LZ1 (70,164.51 and 34,845.46 mg kg^−1^, respectively) and their lowest concentrations in group B. Similarly, K, Mg, Na, and Mn also reached their highest concentrations in group LZ1. Among these elements, Mg and Na reached their lowest concentrations in group LZ2 (13,978.94 and 6229.19 mg kg^−1^, respectively), whereas Mn exhibited its minimum concentration in group L3 (404.17 mg kg^−1^). In contrast, Ca and S exhibited the opposite trend, with their highest concentrations occurring in group B (62,808.94 mg kg^−1^ for Ca and 27,709.13 mg kg^−1^ for S) and their lowest concentrations in groups LZ2 and LZ3, respectively. Ti and P displayed distinct distribution patterns, with both reaching their highest concentrations in group LZ2 (3312.93 and 692.95 mg kg^−1^, respectively) and their lowest concentrations in group B.

Trace elements exhibited marked variations among the four groups, reflecting differences in elemental behavior and matrix composition. Among the relatively abundant trace elements, B reached its highest concentration in group L3 (2572.01 mg kg^−1^), which was more than seven times higher than that in group LZ2 (352.84 mg kg^−1^). Ba reached its highest concentration in group LZ1 (1376.87 mg kg^−1^), compared with its lowest concentration in group B (203.26 mg kg^−1^). Conversely, Sr reached its highest concentration in group B (1996.91 mg kg^−1^) and decreased across the remaining groups, reaching its minimum in group LZ2 (135.97 mg kg^−1^). Most transition metals and heavy metals exhibited a consistent enrichment pattern, with the highest concentrations observed in group LZ1. Specifically, Ni, Cu, Cd, La, Li, Sc, V, and Zn all reached their highest concentrations in group LZ1, consistent with the distribution patterns of the major matrix elements Al and Fe. In contrast, Co, Cr, and Pb reached their highest concentrations in group LZ2, with Pb increasing from 1.33 mg kg^−1^ in group B to 18.05 mg kg^−1^ in group LZ2. The metalloid As and the non-metal Se exhibited their highest concentrations in group B (57.30 and 2.47 mg kg^−1^, respectively) and their lowest concentrations in groups LZ2 and LZ3, respectively. Mo remained relatively stable across all groups, with concentrations ranging from 0.64 mg kg^−1^ in group LZ2 to 1.12 mg kg^−1^ in group LZ1 (Table S1).

### 3.2. Physicochemical Properties and Clay Mineral Characteristics of Attapulgite

The physicochemical properties, including pH and EC, varied among the four groups. All groups were weakly alkaline, with mean pH values ranging from 8.00 to 8.47. Group L3 exhibited the highest pH (8.47), whereas the remaining groups (B, LZ1, and LZ2) showed similar values ranging from 8.00 to 8.11. L3 and L1 exhibited similarly high EC values of 3.70 and 3.69 mS cm^−1^, respectively. In contrast, LZ2 exhibited the lowest EC value (2.64 mS cm^−1^) (Table 1).

Quartz content was highest in group LZ3 (45.7%), approximately three times that in group LZ1 (16.3%). Group LZ2 contained the highest proportions of both K-feldspar (21.5%) and plagioclase (9.0%). In contrast, K-feldspar content was much lower in groups B (2.4%) and LZ3 (3.5%). The distribution of carbonate minerals differed among the four groups. Calcite reached its highest abundance in group B (14.1%) and its lowest abundance in group LZ2 (0.4%). In contrast, dolomite (11.0%) and siderite (2.6%) reached their highest abundances in group LZ2. Hematite (1.5%) was detected only in group B. Amphibole content was highest in group B (28.3%), decreased to approximately 12% in groups LZ1 and LZ2, and was not detected in group LZ3. Gypsum was detected only in group LZ3 at a low abundance (1.1%) (Table 2).

Smectite abundance showed the same trend as the mixed-layer ratio (%S), decreasing in the order of B (17.0%) > LZ1 (16.0%) > LZ3 (14.0%) > LZ2 (13.0%). The I/S mixed-layer phase was most abundant in groups B (32.0%) and LZ3 (28.0%) but decreased to 16.0% in group LZ2. Illite content was identical in groups B and LZ3 (12.0%) and reached its lowest value in group LZ2 (4.0%). Kaolinite content was highest in group LZ1 (25.0%), followed by group LZ2 (16.0%). In contrast, kaolinite content was much lower in groups B (4.0%) and LZ3 (5.0%). Chlorite content varied only slightly among the groups, ranging from 5.0% in group LZ3 to 8.0% in group B. Similarly, the C/S mixed-layer content showed little variation among the groups, ranging from 4.0% to 5.0%. Palygorskite content was highest in group LZ3 (9.0%) and lowest in group LZ2 (3.0%) (Table 3).

### 3.3. Effect of Aqueous Extracts on As Removal from Precipitates and Aqueous Solutions

**Figure 1:**
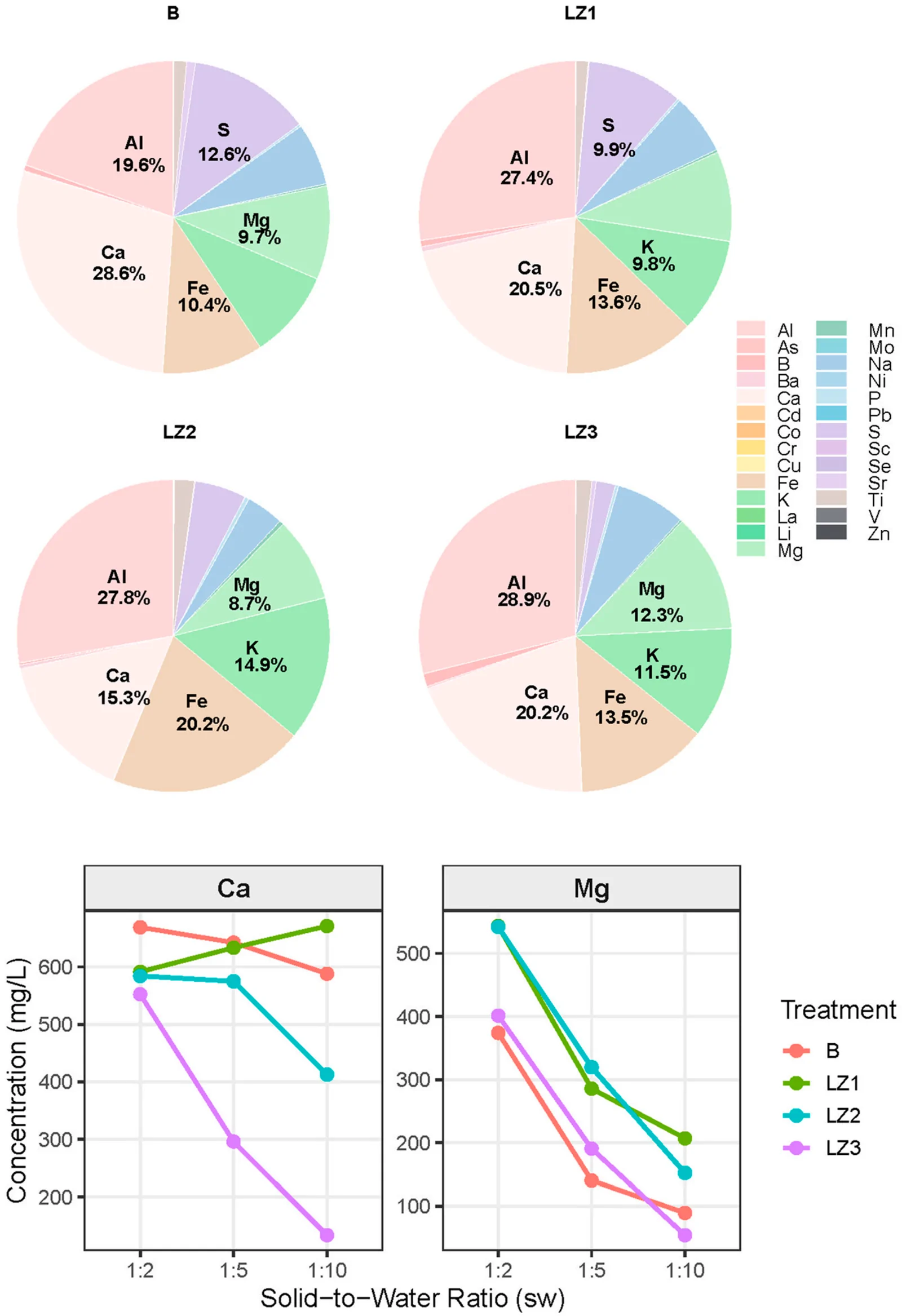
Elemental composition and water-extractable cation dynamics under different treatments. Pie charts presenting the relative abundance (wt.%) of major elements in samples under different amendment treatments (B, LZ1, LZ2, and LZ3). Variations in soluble calcium and magnesium concentrations as a function of the solid-to-water ratio (sw = 1:2, 1:5, and 1:10) across the respective treatments.

**Figure 2:**
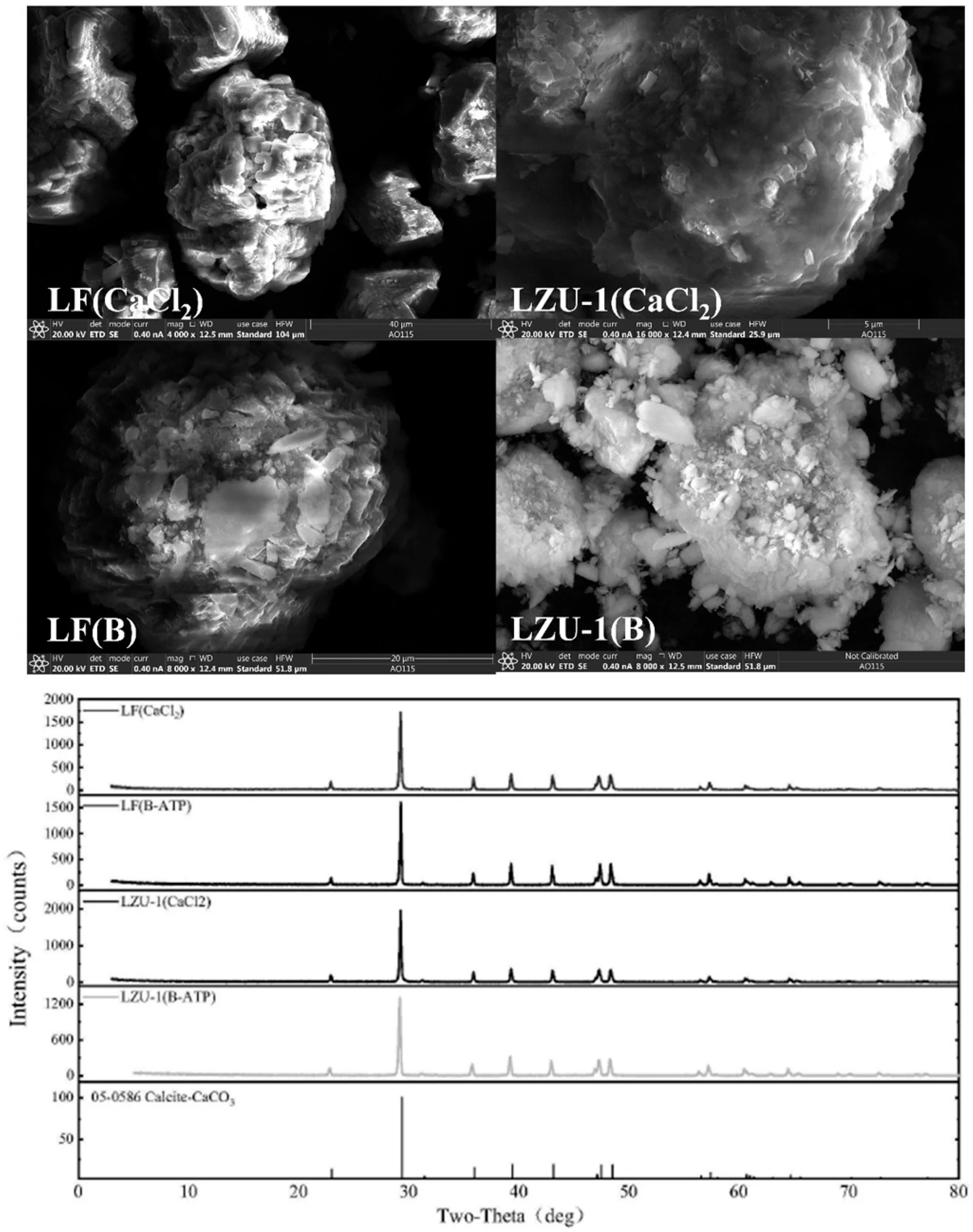
Morphological and mineralogical characterization of the modified substrates. Top panel: Scanning electron microscopy (SEM) micrographs illustrating the surface micro-morphology of LF and LZU-1 matrices treated with CaCl_2_ andttapulgite extracts. Bottom panel: X-ray diffraction (XRD) patterns of the corresponding treated samples compared with the standard reference card of calcite (CaCO_3_, PDF No. 05-0586).

Calcium extraction efficiency from attapulgite varied with the solid-to-liquid ratio (Figure 1). Among the tested solid-to-liquid ratios (1:2, 1:5, and 1:10), the 1:10 ratio produced the highest overall calcium recovery by balancing extract volume and calcium concentration. Although higher clay loadings (1:2 and 1:5) yielded extracts with higher calcium concentrations, the total calcium recovered per unit mass of attapulgite was greatest at the 1:10 ratio. Among the attapulgite samples, LZ3 exhibited the greatest decrease in calcium concentration as the solid-to-liquid ratio decreased, whereas the remaining samples showed relatively stable extraction profiles. Both bacterial strains produced visible white precipitates when cultured in media supplemented with attapulgite aqueous extract and urea. Scanning electron microscopy (SEM) revealed clear morphological differences between precipitates formed using the two calcium sources (Figure 2). When CaCl_2_ was used as the calcium source, both *L. fusiformis* LF and *Enterococcus* LZU-1 produced well-defined rhombohedral crystals characteristic of calcite (Figure 2). In contrast, precipitates formed using the B extract exhibited irregular rhombohedral morphologies.

X-ray diffraction (XRD) analysis showed that the precipitates obtained from both calcium sources consisted predominantly of calcite (Figure 2). Characteristic calcite diffraction peaks corresponding to d-spacings of 3.86, 3.04, 2.49, 2.28, 2.10, and 1.91 Å were observed in precipitates derived from both CaCl_2_ and the B extract. No significant differences in mineral phase composition were observed between precipitates produced using the two calcium sources.

**Figure 3:**
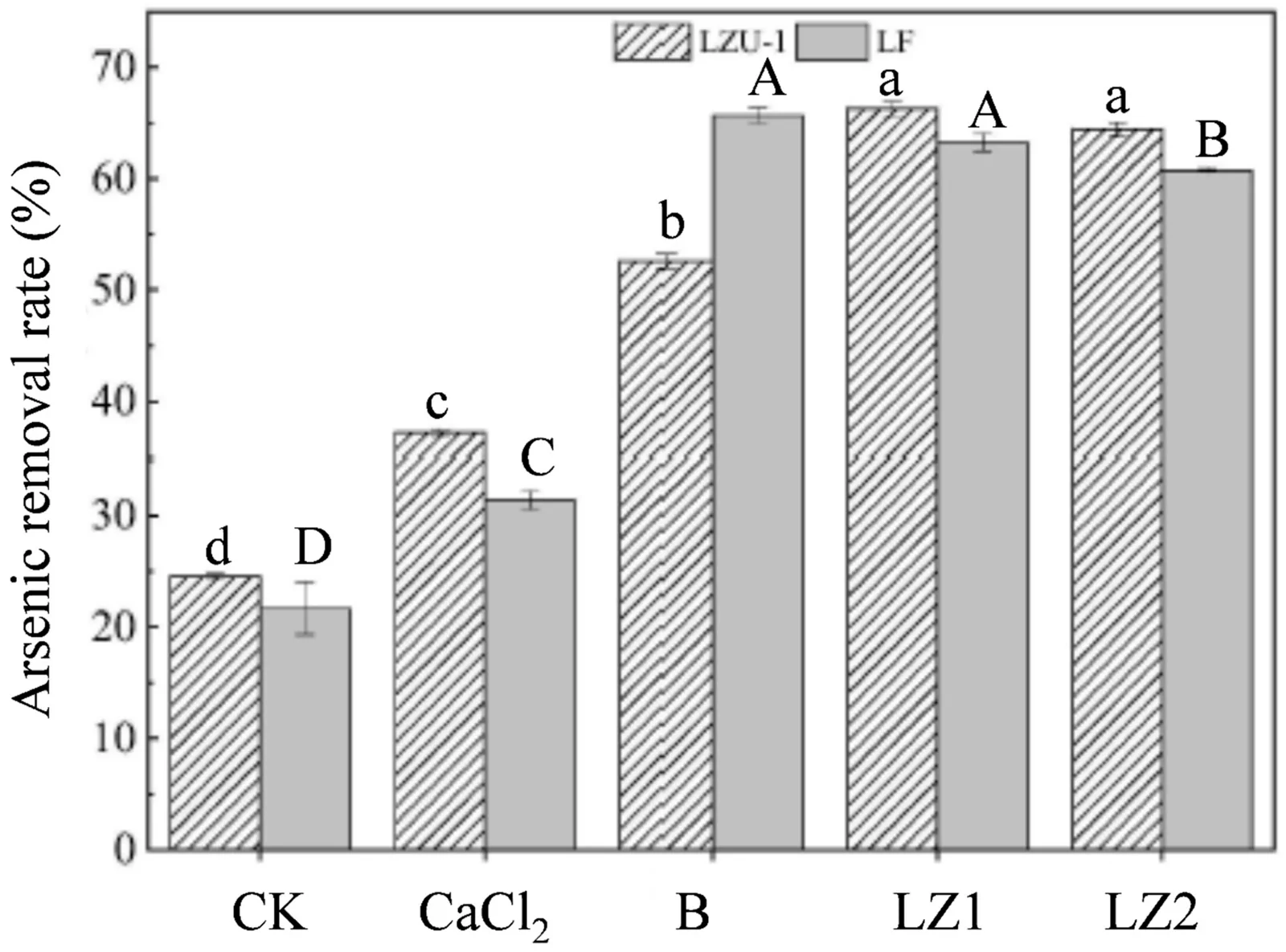
Arsenic removal efficiency across different treatment groups. Arsenic removal rates (%) achieved by LZU-1 and LF matrices under various amendment conditions (CK: control, CaCl_2_, B, LZ1, and LZ2). Error bars represent the standard deviation of replicates (n = 3). Different lowercase letters (for LZU-1) and uppercase letters (for LF) denote statistically significant differences among treatments at the *p* < 0.05 level according to Tukey’s post-hoc test.

The performance of the MICP process driven by the two bacterial strains (LZU-1 and LF) across different calcium sources is illustrated in Figure 3. The experimental data clearly demonstrated that the incorporation of attapulgite aqueous extracts significantly enhanced the immobilization and removal of As from the aqueous phase compared to both the blank control (CK) and the standard chemical calcium control (CaCl_2_). For the blank control group (CK), which lacked an exogenous calcium source, the baseline As removal rates were recorded at approximately 24.5% for strain LZU-1 and 21.8% for strain LF. Upon the addition of 60 mM CaCl_2_, the As removal rates moderately increased to 37.3% for LZU-1 and 31.2% for LF. In striking contrast, the experimental groups utilizing attapulgite clay extracts (B, LZ1, and LZ2) exhibited dramatically superior As removal performance. For strain LZU-1, the removal efficiency advanced to 52.6% in group B, and peaked exceptionally at 66.4% in group LZ1 and 64.3% in group LZ2. For strain LF, a distinct trend was observed: group B achieved its outstanding highest removal rate of 65.8%, while groups LZ1 and LZ2 achieved 63.2% and 60.7%, respectively. These variations indicate a highly strain-specific compatibility and interaction with the diverse mineral elements dissolved from different attapulgite clay sources.

### 3.4. Soil As Remediation and Key Driving Factors

Compared with the control group (CK, pH ≈ 7.1), all remediation treatments significantly increased soil pH (*p* < 0.05). The attapulgite treatment (AT) produced the highest pH (≈8.0), followed by the bacterial treatment (U) and the combined treatment (ATU), both of which ranged from approximately 7.4 to 7.5. The EC of the CK group was 2.6 mS cm^−1^. The bacterial treatment (U) increased the EC to 3.8 mS cm^−1^. The AT treatment resulted in the highest EC value (approximately 7.6 mS cm^−1^; *p* < 0.05). In contrast, the combined treatment (ATU) maintained the EC at 2.8 mS cm^−1^, which was similar to that of the CK group.

**Figure 4:**
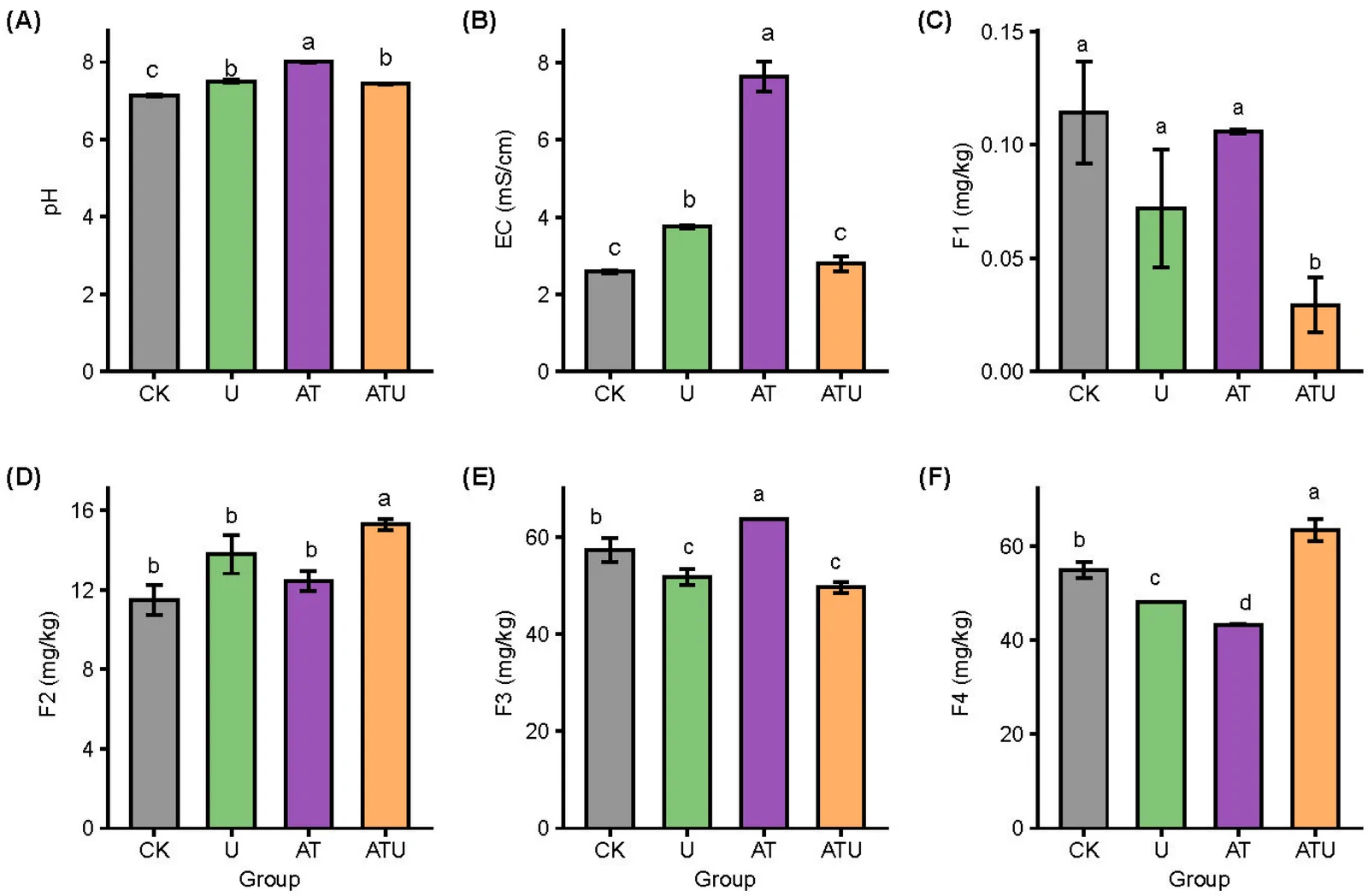
Alterations in soil physicochemical properties and arsenic geochemical fractions. Effects of different treatments (CK, U, AT, and ATU) on soil (**A**) pH, (**B**) electrical conductivity (EC), and (**C**–**F**) the concentration of arsenic distributed in sequential Tessier’s extraction fractions: (**C**) F1 (exchangeable fraction), (**D**) F2 (carbonate bound fraction), (**E**) F3 (iron-manganese oxidized bound fraction), and (**F**) F4 (organic bound fraction). Data are shown as mean ± SD (n = 3), and different lowercase letters indicate significant differences (*p* < 0.05).

The F1 fraction represents the most bioavailable fraction of As. Compared with the CK group (0.115 mg kg^−1^), the U and AT treatments did not significantly affect the F1 fraction (*p* > 0.05). In contrast, the ATU treatment significantly reduced the F1 fraction to approximately 0.03 mg kg^−1^ (Figure 4C), representing the lowest value among all treatments (*p* < 0.05). The F2 fraction in the CK group was 11.5 mg kg^−1^. The AT treatment did not differ significantly from the CK group, whereas both the U and ATU treatments significantly increased the F2 fraction (*p* < 0.05), with the ATU group reaching the highest value (15.3 mg kg^−1^; Figure 4D). For the F3 fraction, the AT treatment produced the highest value (approximately 64 mg kg^−1^; *p* < 0.05). In contrast, the U and ATU treatments significantly decreased the F3 fraction from approximately 57 mg kg^−1^ in the CK group to around 50 mg kg^−1^ (Figure 4E). The F4 fraction showed different responses among the treatments (Figure 4F). Compared with the CK group (55 mg kg^−1^), the F4 fraction decreased to 48 mg kg^−1^ in the U treatment and to 43 mg kg^−1^ in the AT treatment (*p* < 0.05). The ATU treatment significantly increased the F4 fraction to approximately 64 mg kg^−1^, the highest value among all treatments (*p* < 0.05).

**Figure 5:**
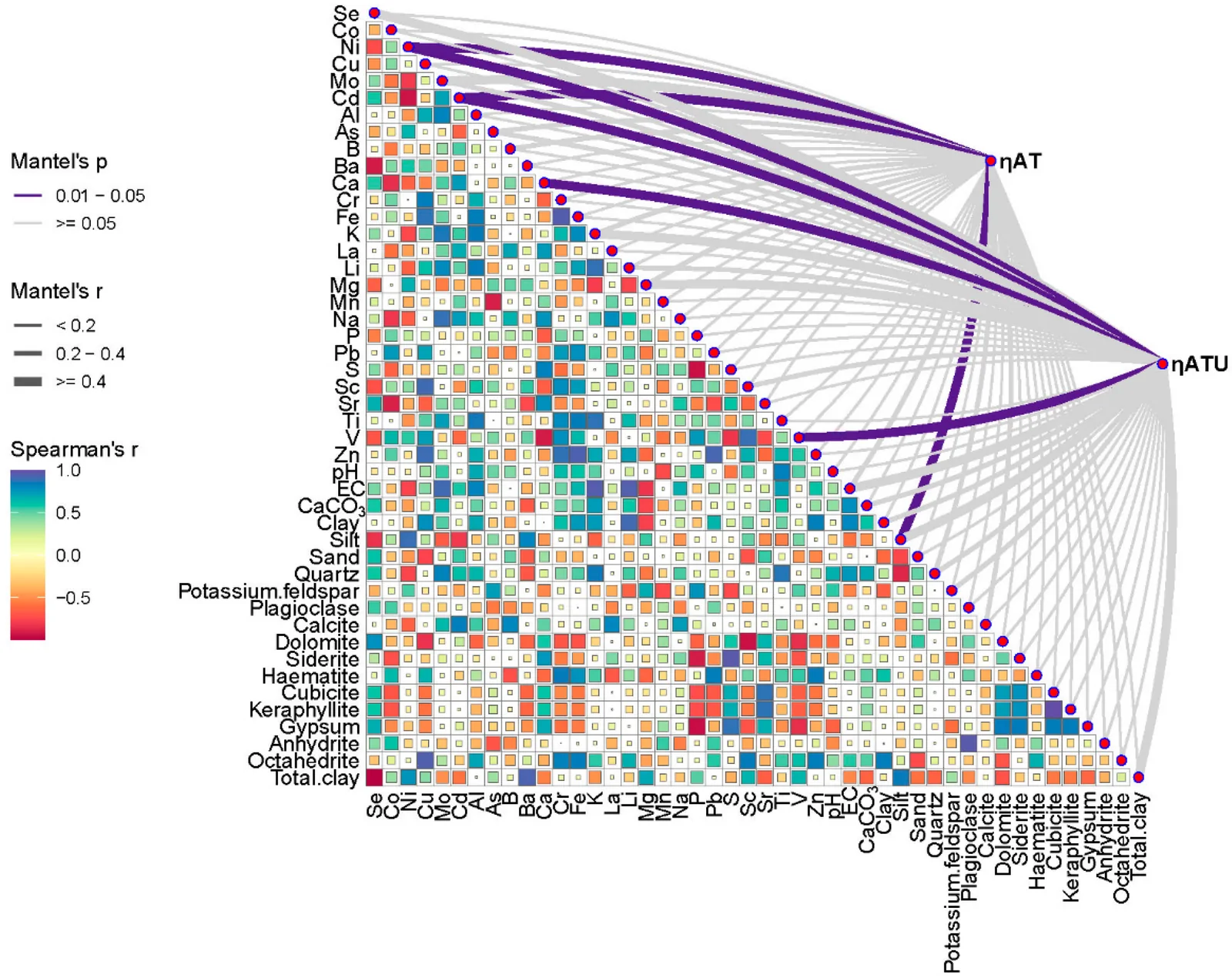
Inter-relationship matrix and Mantel test analysis. The color-scaled heatmap displays Spearman’s rank correlation coefficients (r) among soil elemental contents, physicochemical parameters (pH, EC), and mineral compositions (e.g., calcite, quartz, clay minerals). The overlaying network topology tracks the driving factors influencing the performance indicators (ηAT and ηATU). Line (edge) width corresponds to Mantel’s r statistic (effect size), and line color differentiates the significance levels (Mantel’s *p*-value).

A Mantel test was performed to examine the relationships between environmental variables and As remediation efficiency under the attapulgite (ηAT) and combined attapulgite–bacteria (ηATU) treatments (Figure 5). To identify the key environmental drivers that exert a substantial influence on remediation, a threshold of Mantel’s r > 0.4 (accompanied by *p* < 0.05) was applied. This cutoff was selected because r > 0.4 represents a moderate-to-strong effect size in matrix-level correlations, effectively screening out weak or statistically marginal associations. The remediation efficiency of the attapulgite treatment (ηAT) was significantly associated with both soil chemical composition and soil texture. Specifically, ηAT was significantly correlated with Ni, Cd, and silt content (Mantel’s r ≥ 0.4, *p* < 0.05). In contrast, different environmental variables were associated with the remediation efficiency of the combined treatment (ηATU). Specifically, ηATU was significantly correlated with Ni, Cd, Ca, and V (Mantel’s r ≥ 0.4, *p* < 0.05).

## 4. Discussion

### 4.1. Favorable Calcium-Release Profiles and Beneficial Mineral Constituents of Attapulgite Group B for Enhanced MICP Performance

The effectiveness of MICP for environmental remediation largely depends on the availability of a sustainable, cost-effective, and efficient calcium source [33,34]. The comparative analysis of the four attapulgite (ATP) groups indicates that group B possesses favorable geochemical and mineralogical characteristics for MICP-based remediation.

In addition to its high calcium content, the extraction experiment further highlighted the favorable calcium-release characteristics of group B (Figure 1). Unlike group LZ3, which showed a marked decrease in calcium concentration at a solid-to-water ratio of 1:10, group B maintained a relatively stable calcium-release profile. At a solid-to-water ratio of 1:10, group B maintained a calcium concentration of approximately 600 mg L^−1^, providing high calcium recovery while reducing the amount of attapulgite required. This characteristic may improve the practicality of MICP in large-scale engineering applications by minimizing material input and potential slurry-handling challenges. In addition to calcium availability, the chemical composition of the calcium source is an important consideration for environmental remediation. Group B also contained lower concentrations of major matrix elements, particularly Al and Fe, than the LZ groups. Trace element analysis further showed that group B contained lower concentrations of Pb, Ni, Cu, Cd, La, Sc, V, and Zn than the other ATP groups. The Pb concentration in group B (1.33 mg kg^−1^), for example, was substantially lower than that in group LZ2 (18.05 mg kg^−1^). The relatively low concentrations of these potentially toxic metals may reduce inhibitory effects on ureolytic microorganisms, thereby providing a more favorable environment for MICP.

The crystallization behavior and mineralogical characterization (Figure 2) further support the feasibility of using group B extract as a calcium source for MICP. Both *L. fusiformis* LF and *Enterococcus* LZU-1 produced CaCO_3_ when cultured with the group B extract, indicating that its weakly alkaline pH (8.00–8.11) and chemical composition were suitable for bacterial ureolytic activity. XRD analysis showed that the precipitates derived from the B extract exhibited diffraction patterns consistent with the standard calcite phase (PDF 05-0586), similar to those obtained using CaCl_2_. The characteristic calcite diffraction peaks indicate that the use of attapulgite extract did not alter the dominant mineral phase of the precipitates. The formation of calcite suggests good mineral stability, which is favorable for long-term contaminant immobilization [35]. SEM observations further revealed morphological differences between the two calcium sources. Whereas the CaCl_2_ control produced well-defined rhombohedral crystals, the group B extract generated irregularly aggregated rhombohedral particles. This morphological difference may be associated with co-extracted ions (e.g., Mg and S) and minor impurities present in the attapulgite extract, which can influence crystal nucleation and growth [36,37]. These ions may adsorb onto specific crystal faces during nucleation, thereby modifying surface free energy and influencing the growth rates of different crystal planes [38]. The irregularly aggregated crystal morphology may enhance physical interlocking with soil particles, potentially improving mineral stability and contaminant immobilization [39]. We propose a synergistic immobilization mechanism with three cooperating components: (i) microbial urea hydrolysis generates carbonate alkalinity and elevates pH, driving rapid CaCO_3_ precipitation; (ii) the abundant clay and calcite particles serve as physical scaffolding and heterogeneous nucleation sites, promoting the formation of high-surface-area, defect-rich composite precipitates that efficiently occlude arsenate. We have now measured the total Fe and Mn concentrations in the aqueous phase of the clay suspension (after filtration through 0.45 µm) and found them to be [insert value, e.g., very low: Fe < 0.35 mg/kg, Mn < 1.22 mg/kg/or below detection limit] (Figure S3). This finding confirms that maybe dissolved Fe and Mn species contribute only minimally to the observed As removal. The Fe and Mn originally present in the clay mixture are almost entirely associated with the solid particulate phase (clay mineral lattices and oxide coatings), and their contribution is physical rather than chemical—they are part of the solid matrix that facilitates entrapment and adsorption. This is fully consistent with the physical scaffolding mechanism described above.

### 4.2. Mechanisms of As Immobilization in Aqueous and Soil Systems via Combined Attapulgite and MICP Remediation

The exceptional superiority of the attapulgite clay extracts over pure chemical CaCl_2_ can be elucidated by the complex chemical composition of natural clay extracts. Beyond providing a steady flux of soluble Ca^2+^ ions, the attapulgite extract contains dissolved silicates, aluminum complexes, and micro-crystalline clay fragments. These entities act as highly effective, heterogeneous nucleation sites that accelerate the kinetics of calcite precipitation induced by microbial urea hydrolysis [40]. The co-precipitation of CaCO_3_ along with iron, aluminum, or silicate complexes creates a highly stable crystalline matrix capable of structurally trapping arsenate ions (AsO_4_^3−^) via isomorphic substitution or surface complexation, thereby driving the aqueous As concentration down far more effectively than standard chemical precipitation [41].

The AT treatment resulted in the highest EC value (approximately 7.6 mS cm^−1^; *p* < 0.05). This marked elevation in EC for the AT group reflects a temporary soil salinization effect, which is primarily driven by the rapid dissolution and release of soluble salts and exchangeable alkaline earth cations (such as Ca^2+^ and Mg^2+^) from the mineral structure of attapulgite. However, this potential salinization hazard was effectively mitigated in the combined treatment (ATU). The ATU group maintained a low EC of 2.8 mS cm^−1^, which was statistically similar to that of the CK group (2.6 mS cm^−1^). This stabilization occurs because the active ureolytic bacteria in the ATU system induce the precipitation of calcium/magnesium carbonates (MICP) using the soluble ions released from the attapulgite. By shifting these free ions from the soil solution into stable, insoluble crystalline phases, the combined ATU treatment successfully neutralizes the secondary salinization risk associated with single clay applications, demonstrating its superior environmental compatibility. The combined ATU treatment reduced the F1 fraction to approximately 0.03 mg kg^−1^, the lowest value among all treatments. This decrease in the F1 fraction was accompanied by an increase in the more stable F2 and F4 fractions, indicating a redistribution of As toward less labile forms. The ATU treatment exhibited the highest F2 content (15.3 mg kg^−1^) and the highest F4 content (approximately 64 mg kg^−1^). In contrast, the AT and U treatments resulted in lower F4 contents than the control group. These findings suggest a synergistic effect between attapulgite and MICP bacteria. Attapulgite likely provides abundant adsorption sites for As and a favorable surface for bacterial attachment, whereas microbial-induced calcite precipitation promotes the incorporation or co-precipitation of As into carbonate minerals. The combined action of adsorption and biomineralization therefore contributes to the transformation of labile As into more stable fractions, resulting in greater immobilization efficiency than either treatment alone. Previous studies have shown that arsenate can substitute for carbonate within the calcite crystal lattice, thereby enhancing its long-term immobilization [42]. Although elevated As(V) concentrations may inhibit the transformation of precursor CaCO_3_ phases into calcite, As(V) can also be effectively removed through co-precipitation during calcite crystallization [43]. These mechanisms are consistent with the increased proportions of stable As fractions observed in the ATU treatment.

Mantel analysis further explored the relationships between environmental variables and remediation efficiency (Figure 5). The results suggest that bacterial inoculation modified the environmental factors associated with remediation efficiency. Whereas the remediation efficiency of the AT treatment (ηAT) was significantly associated with Ni, Cd, and silt content, the combined treatment (ηATU) showed significant correlations with Ni, Cd, Ca, and V. These differences suggest that attapulgite may contribute to MICP through mechanisms beyond serving solely as a calcium source. Attapulgite contains various trace elements that may influence microbial activity and mineralization processes [44,45]. The observed correlations with Ni and V may indicate that these elements participate in regulating microbial metabolism during MICP, although their bioavailability was not directly measured in this study [46,47]. Nickel is an essential cofactor for urease and plays a key role in microbial urea hydrolysis during MICP [48]. Vanadium has also been reported to participate in microbial redox metabolism in certain microorganisms, although its role in MICP remains less well established [49].

## 5. Conclusions

In conclusion, this study demonstrates a sustainable, low-cost approach for As immobilization in both aqueous and soil environments by leveraging natural attapulgite clay extract as an alternative calcium source for MICP. Natural Baiyin attapulgite (group B) provides an exceptional, stable calcium-release profile with minimal toxic heavy metal impurities, supporting efficient ureolytic biomineralization and calcite formation for *Lysinibacillus fusiformis* LF and *Enterococcus* LZU-1. In aqueous systems, the clay extract simultaneously supplies dissolved Ca^2+^ and provides an abundance of fine clay fragments, calcite micro-grains, and amorphous Fe/Al/Mn phases. These constituents serve as physical scaffolding and heterogeneous nucleation sites, disrupting ordered calcite growth and driving the formation of irregular, defect-rich clay-calcite-arsenic composite precipitates. This synergistic matrix effect drastically enhances As immobilization via lattice entrapment and encapsulation achieving removal rates of up to 65.8% (LF and Baiyin attapulgite) and significantly outperforming conventional CaCl_2_ treatments. This significantly outperforms conventional CaCl_2_ treatments (31.2–37.3%) and blank controls. In contaminated agricultural soils, the synergistic combination of attapulgite and MICP bacteria successfully drives the transformation of highly bioavailable exchangeable As into stable carbonate- and organic-bound fractions (reducing exchangeable As to ~0.03 mg kg^−1^), while mitigating soil EC to avoid secondary salinization. Environmental variables, particularly trace elements (Ni, V) and soil characteristics (silt, Ca) significantly regulate the remediation efficiency. Overall, utilizing natural attapulgite clay as a green calcium precursor offers a highly economically viable and ecologically compatible strategy for the scalable remediation of As-contaminated water and agricultural resources.

## Figures and Tables

**Table 1:** Physicochemical properties and water-soluble Ca and Mg contents of the four attapulgite clays.

Sample	pH	EC (mS/cm)	Carbonate (%)	Ca (mg/kg)	Mg (mg/kg)
B	8.11	3.05	14.69	5749.99	893.43
LZ1	8.07	3.69	12.57	5240.11	2069.85
LZ2	8.00	2.64	12.91	3459.72	1523.24
LZ3	8.47	3.70	13.30	5234.17	540.64

**Table 2:** Bulk mineral composition of the four attapulgite clays (%).

Sample	Quartz	K-Feldspar	Plagioclase	Calcite	Dolomite	Siderite	Hematite	Amphibole	Gypsum
B	26.7	2.4	6.0	14.1	1.1	0	1.5	28.3	0
LZ1	16.3	12.7	1.8	3.7	0.8	0.4	0	12.1	0
LZ2	26.9	21.5	9.0	0.4	11.0	2.6	0	12.9	0
LZ3	45.7	3.5	5.6	3.2	5.3	0	0	0	1.1

**Table 3:** Clay mineral composition of the four attapulgite clays.

Sample	S (%)	I/S (%)	I (%)	K (%)	C (%)	C/S (%)	Pa (%)	Mixed-Layer Ratio (%)
B	17	32	12	4	8	5	6	17
LZ1	16	25	10	25	7	4	5	16
LZ2	13	16	4	16	6	5	3	13
LZ3	14	28	12	5	5	4	9	14

Note: S denotes montmorillonite, I/S denotes illite-montmorillonite mixed-layer clay, I denotes illite, K denotes kaolinite, C denotes chlorite, C/S denotes chlorite-montmorillonite mixed-layer clay, and Pa denotes attapulgite (also known as palygorskite). The mixed-layer ratio (%S) represents the percentage content of montmorillonite.

## Data Availability

The data relevant to this study are available from the authors on request.

## Supplementary Materials

The following supporting information can be downloaded at: https://www.mdpi.com/article/10.3390/toxics14080678/s1. Table S1. Concentrations of the 27 elements (mg/kg) across different groups. Figure S1. Growth curves of two types of bacteria. Figure S2. The urease production activities of the two types of bacteria. Figure S3. Concentrations of iron (Fe) and manganese (Mn) as a function of solid-to-water ratio under different treatment conditions.

## References

[1] Rehman M.U., Khan R., Khan A., Qamar W., Arafah A., Ahmad A., Ahmad A., Akhter R., Rinklebe J., Ahmad P. (2021). Fate of arsenic in living systems: Implications for sustainable and safe food chains. J. Hazard. Mater..

[2] Shang Y., Li J., Dong W., Arshad S., Zhang X., Shi X., Wu Y., Weng X., Zhang D., Wang D. (2026). Arsenic in groundwater and soil across China: Spatial patterns, drivers, and risks to rice security and human health. Environ. Int..

[3] Al-Makishah N.H., Taleb M.A., Barakat M.A. (2020). Arsenic bioaccumulation in arsenic-contaminated soil: A review. Chem. Pap..

[4] Zhang S., Zhang J., Niu L., Chen Q., Zhou Q., Xiao N., Man J., Ma J., Wei C., Zhang S. (2024). Escalating arsenic contamination throughout Chinese soils. Nat. Sustain..

[5] Chauhan R., Awasthi S., Tiwari P., Upadhyay M.K., Srivastava S., Dwivedi S., Dhankher O.P., Tripathi R.D. (2024). Biotechnological strategies for remediation of arsenic-contaminated soils to improve soil health and sustainable agriculture. Soil Environ. Health.

[6] Banfield J., Barker W.W., Welch S., Taunton A. (1999). Biological impact on mineral dissolution: Application of the lichen model to understanding mineral weathering in the rhizosphere. Proc. Natl. Acad. Sci. USA.

[7] Torres-Aravena Á.E., Duarte-Nass C., Azócar L., Mella-Herrera R., Rivas M., Jeison D. (2018). Can microbially induced calcite precipitation (MICP) through a ureolytic pathway be successfully applied for removing heavy metals from wastewaters?. Crystals.

[8] Li W.-Q., Li Y.-H., Zhu C.-Q., Wang K., Guo X., Zhang M.-C. (2026). Mechanisms of microbially induced calcium carbonate precipitation for simultaneous immobilization of lead–chromium composite contaminants in water. Water Sci. Eng..

[9] Zhang W., Zhang H., Xu R., Qin H., Liu H., Zhao K. (2023). Heavy metal bioremediation using microbially induced carbonate precipitation: Key factors and enhancement strategies. Front. Microbiol..

[10] Ji G., Huan C., Zeng Y., Lyu Q., Du Y., Liu Y., Xu L., He Y., Tian X., Yan Z. (2024). Microbiologically induced calcite precipitation (MICP) in situ remediated heavy metal contamination in sludge nutrient soil. J. Hazard. Mater..

[11] Dong H., Huang L., Zhao L., Zeng Q., Liu X., Sheng Y., Shi L., Wu G., Jiang H., Li F. (2022). A critical review of mineral–microbe interaction and co-evolution: Mechanisms and applications. Natl. Sci. Rev..

[12] Rajasekar A., Wilkinson S., Moy C.K.S. (2021). MICP as a potential sustainable technique to treat or entrap contaminants in the natural environment: A review. Environ. Sci. Ecotechnol..

[13] Achal V., Pan X. (2014). Influence of calcium sources on microbially induced calcium carbonate precipitation by *Bacillus* sp. CR2. Appl. Biochem. Biotechnol..

[14] Feng Q., Song Y., Lu C., Fang H., Huang Y., Chen L., Song X. (2023). Feasible utilization of waste limestone as a calcium source for microbially induced carbonate precipitation (MICP). Fermentation.

[15] Fu F., Wang Q. (2011). Removal of heavy metal ions from wastewaters: A review. J. Environ. Manag..

[16] Gowthaman S., Chen M., Nakashima K., Kawasaki S. (2021). Effect of scallop powder addition on MICP treatment of amorphous peat. Front. Environ. Sci..

[17] Gowthaman S., Yamamoto M., Nakashima K., Ivanov V., Kawasaki S. (2021). Calcium phosphate biocement using bone meal and acid urease: An eco-friendly approach for soil improvement. J. Clean. Prod..

[18] Joseph L., Jun B.-M., Flora J.R.V., Park C.M., Yoon Y. (2019). Removal of heavy metals from water sources in the developing world using low-cost materials: A review. Chemosphere.

[19] Sen R. (2025). BMC-014—Sugarcane bagasse hydrolysate as an inexpensive carbon source for microbially induced calcium carbonate precipitation: A sustainable approach towards construction biotechnology. New Biotechnol..

[20] Wang Y., Feng Y., Jiang J., Yao J. (2018). Designing of recyclable attapulgite for wastewater treatments: A review. ACS Sustain. Chem. Eng..

[21] Du X., Wang R., Liu Y., Yang H., Feng Q., Bai G., Zhou Q., Wu Z., Zhang Y. (2025). Study on the mechanisms of water ecological remediation integrated by attapulgite and submerged macrophyte. Innov. Geosci..

[22] Turan V. (2019). Confident performance of chitosan and pistachio shell biochar on reducing Ni bioavailability in soil and plant plus improved the soil enzymatic activities, antioxidant defense system and nutritional quality of lettuce. Ecotoxicol. Environ. Saf..

[23] Wang R., Zhu J., Li B., Liu Y., Fang Q., Bai G., Tang Y., He F., Zhou Q., Wu Z. (2023). Effects of attapulgite on the growth status of submerged macrophytes Vallisneria spiralis and sediment microenvironment. J. Environ. Manag..

[24] Chen H., Ai Y., Jia Y., Li J., Gu M., Chen M. (2022). Effective and simultaneous removal of heavy metals and neutralization of acid mine drainage using an attapulgite-soda residue based adsorbent. Sci. Total Environ..

[25] Gu S., Kang X., Wang L., Lichtfouse E., Wang C. (2019). Clay mineral adsorbents for heavy metal removal from wastewater: A review. Environ. Chem. Lett..

[26] Ning X., Yang M., Li C., He L., Long S., Wang S. (2026). Bioremediation of arsenic in calcareous soil by microbial-induced calcium carbonate precipitation and attapulgite in arid northwest China. J. Clean. Prod..

[27] Wu Y., Wang S., Ning X., Yang M., Liu M., Zang F., Nan Z. (2021). A promising amendment for the immobilization of heavy metal(loid)s in agricultural soil, northwest China. J. Soils Sediments.

[28] Yang M., Wang S., Liu M., Ning X., Wu Y., Nan Z. (2023). Dose relationships and interactions of four materials and MICP technology in simultaneously reducing the exchangeable parts of As, Pb, and Cd in multiple contaminated soils. J. Soils Sediments.

[29] Liu Z., Ning X., Long S., Wang S., Li S., Dong Y., Nan Z. (2024). Arsenic and cadmium simultaneous immobilization in arid calcareous soil amended with iron-oxidizing bacteria and organic fertilizer. Sci. Total Environ..

[30] Long S., Ning X., Wang S., Xu J., Wu Y., Liu Z., Nan Z. (2023). Remediation of arsenic-contaminated calcareous agricultural soils by iron-oxidizing bacteria combined with organic fertilizer. Environ. Sci. Pollut. Res..

[31] Zhao R., Ning X., Long S., Dong Y., He L., Wang W., Gao Y., Sun C., Wang X., Miao R. (2026). Pollution accumulation, sources, and risk assessment of potentially toxic elements in the soil-walnut system: Insights from over 50 years of wastewater irrigation in a lead/zinc smelting region. Environ. Geochem. Health.

[32] Ning X., He L., Long S., Wang S. (2025). Bioavailability, migration and driving factors of As, Cd and Pb in calcareous soil amended with organic fertilizer and manganese oxidizing bacteria in arid northwest China. J. Hazard. Mater..

[33] Jin S., Meng L., Ma Y., Yu Y. (2025). Research progress and applications of microbially induced calcium carbonate precipitation (MICP) in engineering and environment. Total Environ. Eng..

[34] Xiao R., Jiang G., Chai W., Jin Z., Du R., Khan M., Liu Z., Yin H., Xu L. (2025). Microbially induced calcium carbonate precipitation (MICP): Bibliometric analysis, research hotspot evolution, and mechanistic insights (2005–2024). Water.

[35] Beauvais M., Sanchez Monserrate B., Mi J., Rossini A., Parise J., Chapman K. (2025). Non-stoichiometry governs the pathway from amorphous to crystalline calcium carbonate. J. Am. Chem. Soc..

[36] Hao Z., Su Y., Liu S., Zhang X. (2024). Roles of bacteria and extracellular polymeric substance in calcium carbonate formation: Insights from the effects of calcium source and deposition rate on nucleation. Biochem. Eng. J..

[37] Yu J., Wang K., Yang P., Li M., Dong B., Jin Z., Hong S., Ma H. (2024). Simulation of calcium carbonate nucleation processes in confined C-S-H nanopores with different calcium-silicon ratios. Constr. Build. Mater..

[38] Liu Y., Xiao M., Huang X., Park J., Hoffman M.E., Feng Y., An A.K., Li Q., Hoek E.M.V., Jassby D. (2025). Mitigating CaCO_3_ crystal nucleation and growth through continuous ion displacement via alternating electric fields. Nat. Commun..

[39] Ding W., Sun L., Qi Z., Li S., Filipović V., Wu X., He H. (2025). Conservation tillage enhances both organic and inorganic carbon in dryland: Insights from a 20-year field experiment and meta-analysis. Agric. Ecosyst. Environ..

[40] Clarà Saracho A., Haigh S.K., Hata T., Soga K., Farsang S., Redfern S.A.T., Marek E. (2020). Characterisation of CaCO_3_ phases during strain-specific ureolytic precipitation. Sci. Rep..

[41] Zhang T., Lai S., Bai H., Wang Z., Miao Y., Guo H., Zhao Y. (2025). Phase-selective in situ formation of polymorphic FeO(OH) species for optimized arsenic coprecipitation on mechanically activated carbonate minerals. Sep. Purif. Technol..

[42] Catelani T., Perito B., Bellucci F., Lee S.S., Fenter P., Newville M., Rimondi V., Pratesi G., Costagliola P. (2018). Arsenic uptake in bacterial calcite. Geochim. Cosmochim. Acta.

[43] Fukushi K., Munemoto T., Sakai M., Yagi S. (2011). Monohydrocalcite: A promising remediation material for hazardous anions. Sci. Technol. Adv. Mater..

[44] Cao Y., Yang H., Liu Y., Kong F., Zhu Y., Chen Y., Zhu K., Yang Z. (2026). Attapulgite-modified organic compost effectively reduces soil nutrient loss and enhances microbial interactions. J. Environ. Sci..

[45] Chen Y., Tang P., Li Y., Chen L., Jiang H., Liu Y., Luo X. (2022). Effect of attapulgite on heavy metals passivation and microbial community during co-composting of river sediment with agricultural wastes. Chemosphere.

[46] Duan W., Niu Q., Xu X., Li W., Fu D. (2016). Influence of attapulgite addition on the biological performance and microbial communities of submerged dynamic membrane bioreactor. J. Water Reuse Desalin..

[47] Yan Y., Li Y., Liu C., Li J., Qin J., Liang X., Wang Q., Zou Q., Wang X. (2025). La/Fe/Al co-modified attapulgite for controlling sediment internal phosphorus release: Efficacy, mechanisms, dosing strategy effect, and microbial community response. J. Environ. Chem. Eng..

[48] Mazzei L., Musiani F., Ciurli S. (2020). The structure-based reaction mechanism of urease, a nickel dependent enzyme: Tale of a long debate. J. Biol. Inorg. Chem..

[49] Wang S., Wang M., Chen Y., Zhang B. (2026). Single strain mediated biogeochemical cycling of vanadium during redox fluctuations. J. Hazard. Mater..

