# Supplementary material for: Leachates of Calcium-Rich Phases from Attapulgite Clay as a Sustainable Calcium Source for Microbially Induced Carbonate Precipitation: Enhanced Biomineralization and Arsenic Immobilization
Source: Toxics. 2026 Jul 31;14(8):678. doi: 10.3390/toxics14080678 (PMC13517713; doi:10.3390/toxics14080678)
Supplement: Supplementary file 1 [file toxics-14-00678-s001.zip › toxics-4439646-supplementary.pdf]

Leachates of calcium-rich phases from attapulgite clay as a sustainable calcium source for microbially induced carbonate precipitation: enhanced biomineralization and arsenic immobilization

Lei Wang[a], Xiang Ning[b], Meng Yang[b], Shengli Wang[b,*]

a Gansu Nonferrous Engineering Survey Design and Research Institute, Lanzhou 730000, China

b College of Earth and Environmental Sciences, Lanzhou University, Lanzhou 730000, China

*: Corresponding author: Shengli Wang, E-mail address: wangshengl@lzu.edu.cn

Table S1. Concentrations of the 27 elements (mg/kg) across different groups.

Element Category	Element	Group B	Group L1	Group L2	Group L3	Distribution Pattern (Max → Min)
**Macro-elements**	**Al**	42918.25	70164.51	44588.64	55465.29	L1 > L3 > L2 > B
	**Ca**	62808.94	52542.37	24583.74	38829.39	B > L1 > L3 > L2
	**Fe**	22913.50	34845.46	32468.17	25997.05	L1 > L2 > L3 > B
	**K**	20078.42	25074.78	23981.39	22119.65	L1 > L2 > L3 > B
	**Mg**	21230.99	23781.16	13978.94	23559.82	L1 > L3 > B > L2
	**Mn**	509.03	694.42	673.36	404.17	L1 > L2 > B > L3
	**Na**	14061.31	16104.19	6229.19	13881.09	L1 > B > L3 > L2
	**P**	517.11	629.61	692.95	620.38	L2 > L1 > L3 > B
	**S**	27709.13	25448.65	8545.54	3847.86	B > L1 > L2 > L3

	**Ti**	2870.72	3235.29	3312.93	3172.08	L2 > L1 > L3 > B
**Trace Elements**	**B**	1312.74	1657.78	352.84	2572.01	L3 > L1 > B > L2
	**Ba**	203.26	1376.87	608.72	403.06	L1 > L2 > L3 > B
	**Sr**	1996.91	256.73	135.97	833.83	B > L3 > L1 > L2
	**Se**	2.47	1.59	2.38	1.59	B > L2 > L1 > L3
	**Co**	7.78	10.03	12.14	6.84	L2 > L1 > B > L3
	**Ni**	30.53	31.69	28.90	16.91	L1 > B > L2 > L3
	**Cu**	17.30	32.83	24.11	17.32	L1 > L2 > L3 > B
	**Mo**	1.01	1.12	0.64	1.12	L1 $\approx$ L3 > B > L2
	**Cd**	0.23	0.25	0.24	0.22	L1 > L2 > B > L3
	**As**	57.30	36.04	25.00	34.69	B > L1 > L3 > L2

	**Cr**	46.22	59.70	62.86	46.90	L2 > L1 > L3 > B
	**La**	17.49	31.95	10.70	20.62	L1 > L3 > B > L2
	**Li**	30.35	59.75	35.68	32.94	L1 > L2 > L3 > B
	**Pb**	1.33	13.09	18.05	6.15	L2 > L1 > L3 > B
	**Sc**	6.35	10.57	7.49	7.48	L1 > L2 > L3 > B
	**V**	73.62	97.81	96.84	73.28	L1 > L2 > B > L3
	**Zn**	51.02	85.72	83.10	51.85	L1 > L2 > L3 > B

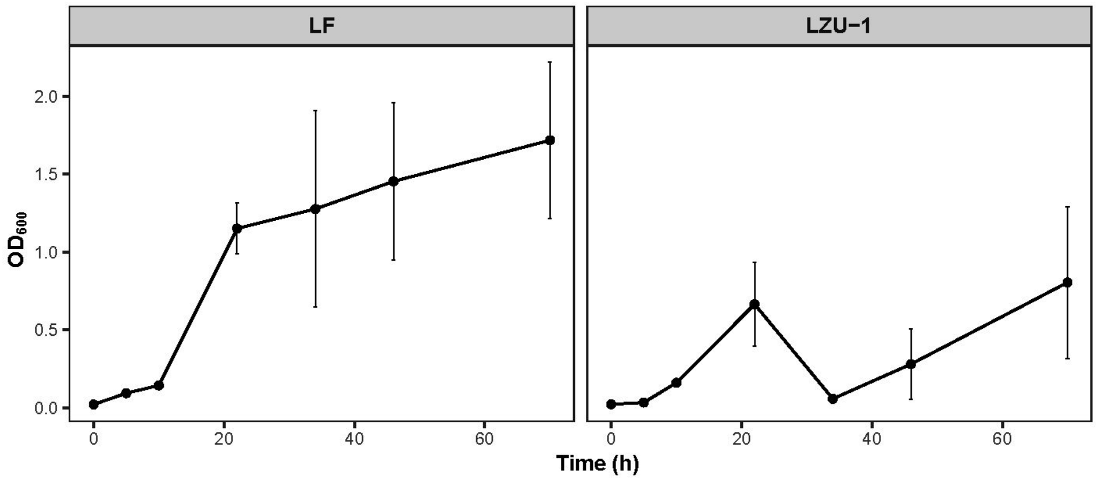

Figure S1. Growth curves of two types of bacteria.

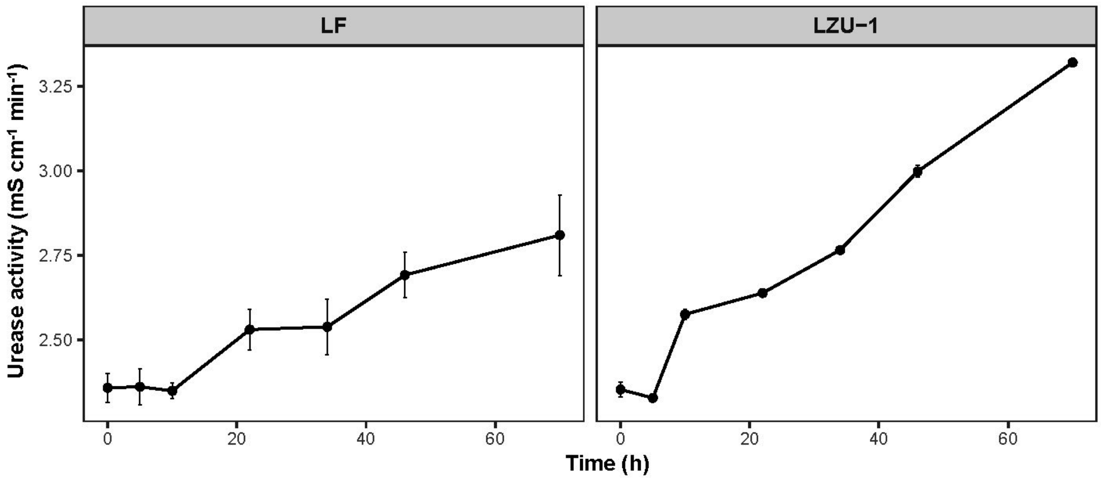

Figure S2. The urease production activities of the two types of bacteria.

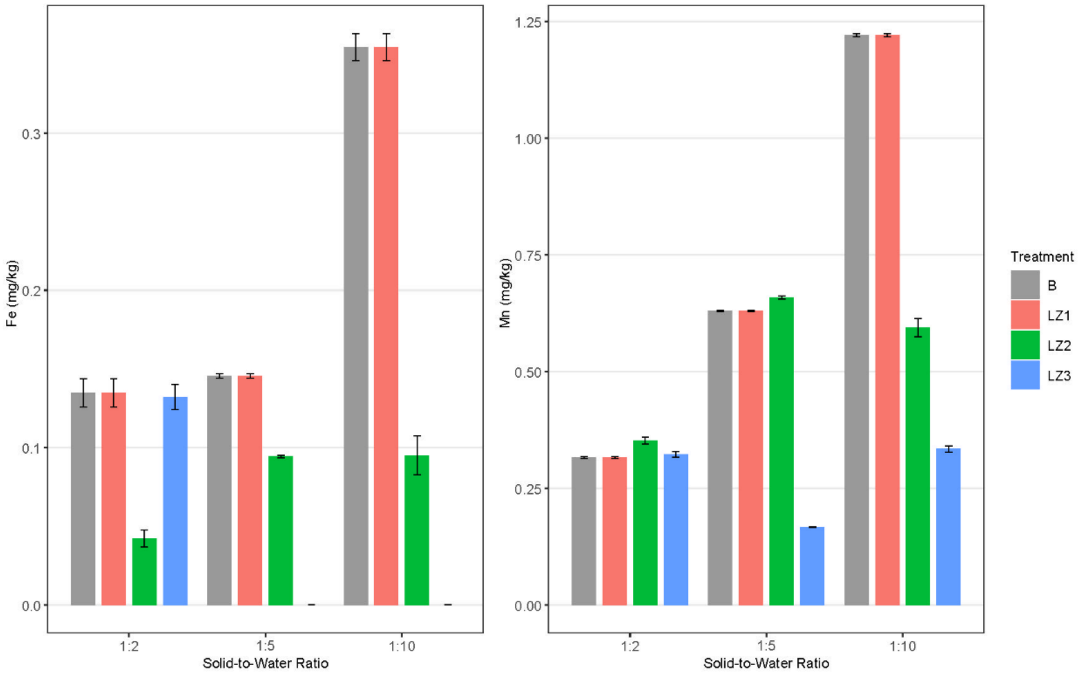

Figure S3. Concentrations of iron (Fe) and manganese (Mn) as a function of solid-to-water ratio under different treatment conditions.
